# Soil microbial abundance was more affected by soil depth than the altitude in peatlands

**DOI:** 10.3389/fmicb.2022.1068540

**Published:** 2022-11-09

**Authors:** Meiling Zhao, Ming Wang, Yantong Zhao, Nanlin Hu, Lei Qin, Zhibin Ren, Guodong Wang, Ming Jiang

**Affiliations:** ^1^Key Laboratory of Wetland Ecology and Environment, Northeast Institute of Geography and Agroecology, Chinese Academy of Sciences, Changchun, China; ^2^College of Resources and Environment, University of Chinese Academy of Sciences, Beijing, China; ^3^State Environmental Protection Key Laboratory of Wetland Ecology and Vegetation Restoration, Institute for Peat and Mire Research, Northeast Normal University, Changchun, China

**Keywords:** microbial abundance, altitudinal gradient, depth gradient, peatland carbon dynamics, co-occurrence network

## Abstract

Soil microbial abundance is a key factor to predict soil organic carbon dynamics in peatlands. However, little is known about the effects of altitude and soil depth and their interaction on soil microbial abundance in peatlands. In this study, we measured the microbial abundance and soil physicochemical properties at different soil depths (0–30 cm) in peatlands along an altitudinal gradient (from 200 to 1,500 m) on Changbai Mountain, China. The effect of soil depth on soil microbial abundance was stronger than the altitude. The total microbial abundance and different microbial groups showed the same trend along the soil depth and altitudinal gradients, respectively. Microbial abundance in soil layer of 5–10 cm was the highest and then decreased with soil depth; microbial abundance at the altitude of 500–800 m was the highest. Abiotic and biotic factors together drove the change in microbial abundance. Physical variables (soil water content and pH) and microbial co-occurrence network had negative effects on microbial abundance, and nutrient variables (total nitrogen and total phosphorus) had positive effects on microbial abundance. Our results demonstrated that soil depth had more effects on peatland microbial abundance than altitude. Soil environmental change with peat depth may lead to the microorganisms receiving more disturbances in future climate change.

## Introduction

Soil microorganisms are a vital part of peatland ecosystems and play a critical role as a “carbon pump” in the process of organic matter decomposition (Liang et al., 2020; Li J. et al., 2022). Ecologists have tried to comprehend the patterns of soil microbial communities along the environmental gradient. With the increasing awareness of the significance of microbial participation in the carbon cycle process and the development of biomarker technology, the pattern of soil microorganisms along the altitudinal and depth gradients has attracted scholars’ attention (Wang et al., 2019; Looby and Martin, 2020). However, existing research so far have not reached the same conclusion. Some studies indicated that the altitude had a stronger impact on the community composition of microbes than soil depth (Li J. et al., 2022), while other studies suggested that soil microbial community activity and composition was more depended on soil depth (Dove et al., 2021; Lamit et al., 2021; Zhao H. et al., 2021). Moreover, several studies demonstrated the complex interaction between altitude and depth had significantly influenced soil microbial abundance and their ratios (Xu et al., 2022). These examples suggest the ongoing debate on the pattern of microbial abundance and communities along the altitudinal and depth gradients. The reason is that these studies focus on different ecosystems or vegetation zones, little is known about the pattern within one ecosystem.

Environmental conditions could affect the distribution of microorganisms in peatlands. For example, soil pH, soil water condition, and dissolved organic carbon (DOC) were found to be major factors influencing the biogeographic patterns of microorganisms (Rousk et al., 2010; Wagner et al., 2015; Wang et al., 2021), and soil nutrients including total nitrogen and total phosphorus significantly affected soil microbial abundance (Fenner and Freeman, 2011). However, the importance of the internal interaction of microorganisms in regulating the microbial abundance has been ignored. In fact, biotic variables (e.g., microbial co-occurrence network) are also supposed to be an important factor influencing the spatial pattern of soil microorganisms (Fan et al., 2017). The symbiosis, predation, and competition relationships between soil microorganisms formed a complex microbial ecological interaction network (Faust and Raes, 2012). The co-occurrence network has been widely used in forest, farmland and other ecosystems to evaluate microbial interactions (Fan et al., 2017; Tu et al., 2020; Xie et al., 2020). However, there are limited data about the living microbial lipid co-occurrence network along the altitudinal and depth gradients in peatlands.

The Changbai Mountain in northeastern China are exceedingly vulnerable to climate change, and the peatlands are widely distributed in this region (Wang et al., 2018; Zhao M. L. et al., 2021), which provides an ideal place to study the pattern in microbial abundance along the altitudinal and depth gradients. In the present study, we set four altitudinal gradients and six depth gradients in peatlands in the Changbai Mountain to understand the change in microbial abundance and their responses to abiotic and biotic factors along the altitudinal and depth gradients.

## Materials and methods

### Study area

The Changbai Mountain is located in Jilin Province, northeastern China (Zhao et al., 2022). The peatlands in the Changbai Mountain are dominated by sedges (Bao et al., 2010; Wang et al., 2018). The climate in this region is a typical continental monsoon climate; the mean annual temperature (MAT) in the study area ranges from −0.2°C to 3.9°C, and the mean annual precipitation (MAP) ranges from 580 to 770 mm in the study area (Zhao et al., 2022).

### Sample collection

**Figure 1:**
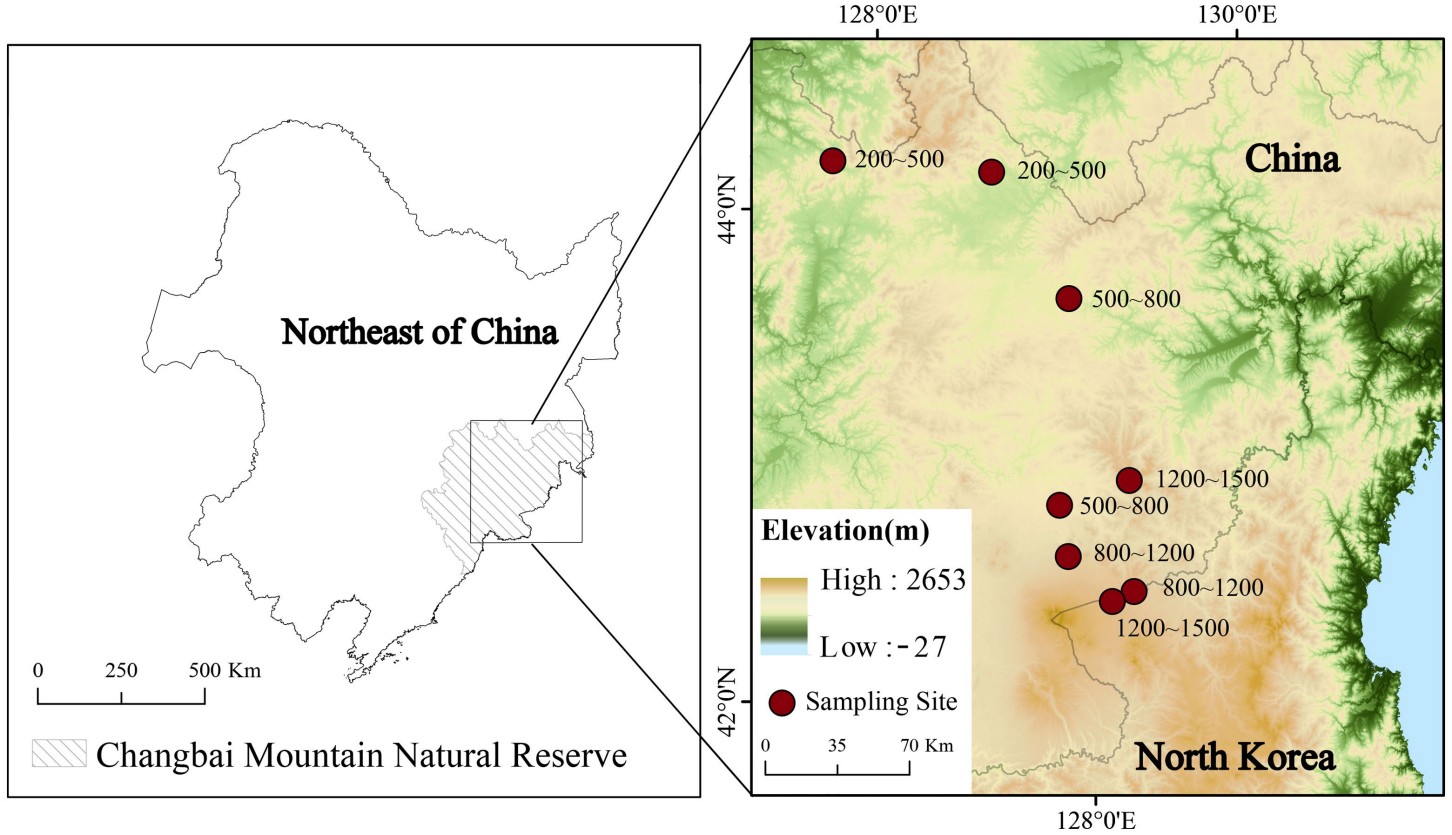
Site locations of the sedge peatlands along an altitudinal gradient on Changbai Mountain, China.

Samples were collected in July 2020. Soil cores at 0–30 cm depth with an interval of 5 cm were collected in four altitude gradients (200–500, 500–800, 800–1,200 and 1,200–1,500 m; Figure 1). Two peat samples were collected in each altitudinal gradient. Peat samples were kept at −20°C immediately after collection. Each sample was separated into two subsamples. One was used for the measurement of soil water content (SWC), and the other was freeze-dried and sieved for the measurement of extracted microbial lipids and soil physicochemical properties.

### Phospholipid fatty acids analysis

The analysis of microbial abundance was used by phospholipid fatty acids (PLFAs) technology. The extraction and separation of PLFAs were performed followed the Bligh Dyer method (Bligh and Dyer, 1959). The sample test method was described by Zhao M. L. et al. (2021). The detected compounds were identified in the MIDI library (MIDI, Inc., Newark, United States) (Zhang et al., 2019). The PLFAs were used as the microbial biomarkers according to Joergensen (2021). The gram-positive bacteria (G+) were the sum of the abundances of Firmicutes and Actinobacteria, the G+ and gram-negative bacteria (G-) belong to bacteria (B). The total microbial abundance was the sum of the abundances of bacteria, fungi (F) and unspecific microbial biomarkers.

### Soil physicochemical property measurement

Soil water content (SWC) was measured by the gravimetric method. Soil pH was measured by a potentiometric test with a soil to water ratio of 1:10. The total organic carbon (TOC) and dissolved organic carbon (DOC) were detected on a TOC analyzer (Shimazu, Japan). The total phosphorus (TP) and total nitrogen (TN) were determined by an automated analyzer (Smartchem140, AMS-Alliance, and French; Lu, 2000).

### Microbial co-occurrence network analysis

Co-occurrence network was used to show microbial biomarker interactions, the analysis was visualized using Gephi v.0.9.1. Each node means one microbial lipid and each edge means a strong relationship between two nodes. The topology of the co-occurrence networks was assessed referred to previous studies (Tu et al., 2020; Li J. et al., 2022). Briefly, average degree indicates the complexity of the co-occurrence network. Average path length indicates the distance between any two members of the co-occurrence network. A higher average degree indicates a higher complexity of the network, a shorter average path length suggests a stronger correlation between members.

### Data analysis

Two-way ANOVAs were used to examine the main effect of altitude and soil depth and their interaction on peat physicochemical properties and soil microbial abundance. Redundancy analysis (RDA) was conducted to analyze the relationship of microbial abundance to peat physicochemical properties. Variation decomposition analysis (VDA) was used to analyze the effects of peat physicochemical properties and microbial co-occurrence networks on soil total microbial abundance. The physical (pH and SWC) and nutrient (DOC, TN, TOC, and TP) variables were used as abiotic factors and the microbial ecological interaction network was used as the biotic factor in the prediction model. Based on previous studies, the microbial co-occurrence networks are represented by the first two axes’ scores of the principal component analysis for microbial community composition (Purahong et al., 2016). A positive coefficient of VDA indicates a positive effect on the prediction of the total microbial abundance, and a negative coefficient suggests the opposite (Gross et al., 2017). The data were log_10_-transformed to conform to normality and homogeneity of variance. The analyses were performed using SPSS 21.0, Canoco 5.0, Origin 29.0, and R 4.1.1 with the packages vegan (Oksanen et al., 2020), MuMIn (Bartoń, 2022), performance (Lüdecke et al., 2021), ggplot2 (Wickham, 2016), and ggh4x (van den Brand, 2021).

## Results

### Soil microbial abundance changes with depth and altitude

**Figure 2:**
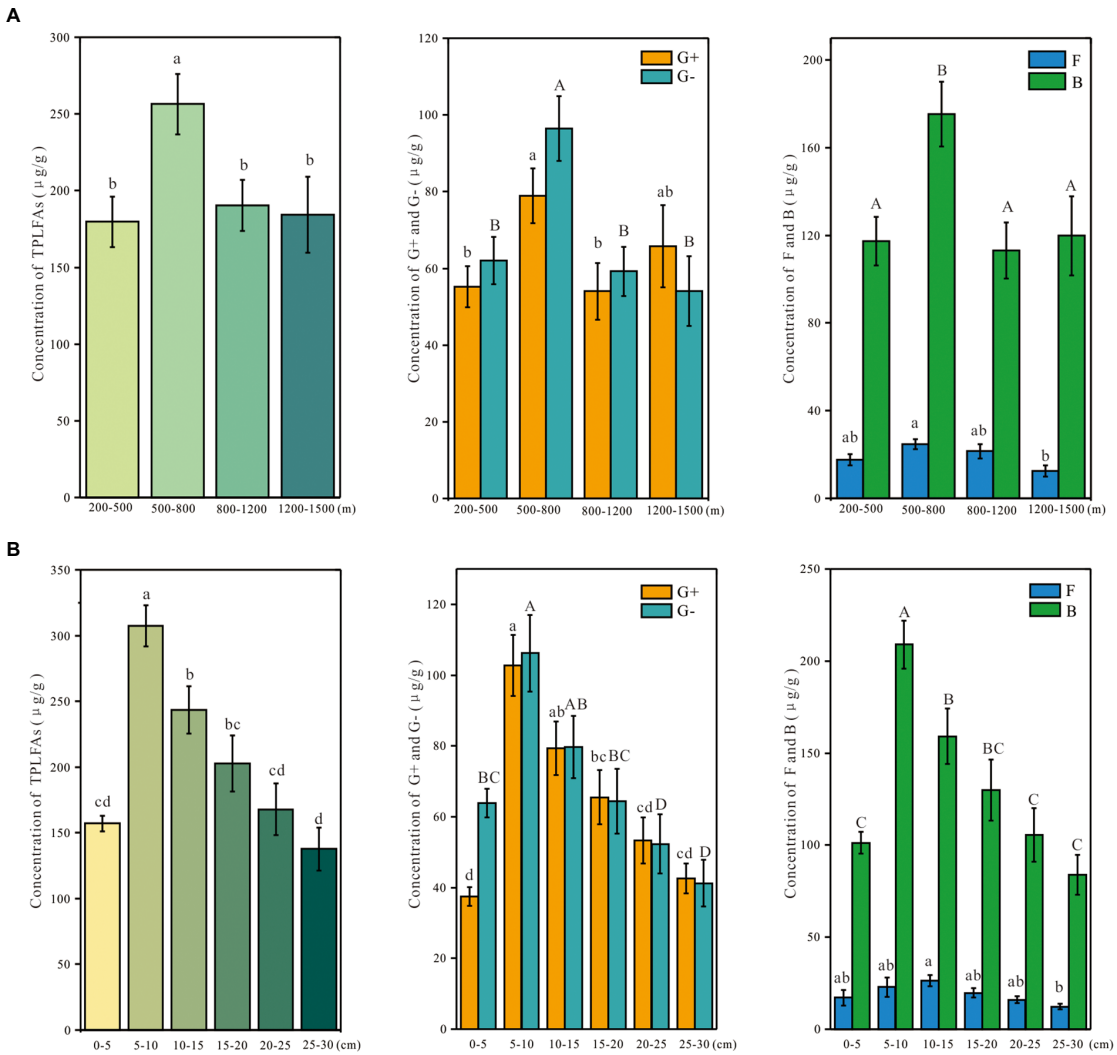
Concentration of various microbial groups based on the phospholipid fatty acids along the depth **(A)** and altitudinal **(B)** gradient on Changbai Mountain, China. Values were shown as Means ± SE. Different letters suggest significant differences among study sites based on one-way ANOVA and Tukey’s test (*p* < 0.05). TPLFAs: total phospholipid fatty acids abundance; G+: gram-positive bacteria; G−: gram-negative bacteria; F: fungi; B: bacteria.

Soil depth and altitude significantly affected the total microbial abundance (Table 1). The effect of soil depth on the abundance of the microbial group was stronger than the altitude (Table 1). The soil layer of 5–10 cm had the highest total microbial concentration, which then decreased with soil depth (Figure 2A). The concentration of total microbial PLFAs was higher at 500–800 m than at other altitudes (Figure 2B). The concentrations of G+, G−, and F showed a similar trend with the total microbial concentration (Figure 2).

**Table 1:** A summary of analysis of variance (ANOVA) on the effects of altitude and soil depth for soil physicochemical properties and microbial groups.

Variable	Altitude		Depth		Altitude × depth	
	*f* value	Value of *p*	*f* value	Value of *p*	*f* value	Value of *p*
SWC	4.04	0.019*	1.702	0.173	0.776	0.690
TOC	2.03	0.136	1.813	0.148	0.622	0.828
DOC	2.759	0.064	40.149	<0.001**	1.181	0.348
TN	1.22	0.324	68.85	<0.001**	1.376	0.236
TP	1.043	0.391	0.676	0.646	0.824	0.644
pH	0.829	0.491	5.84	0.001**	1.039	0.454
Firmicutes	5.731	0.004**	17.948	<0.001**	1.739	0.110
Actinobacteria	5.428	0.005**	12.255	<0.001**	0.871	0.601
G+ bacteria	6.273	0.003**	18.868	<0.001**	1.418	0.216
G− bacteria	11.777	<0.001**	10.928	<0.001**	0.663	0.793
Fungi	4.29	0.015*	5.696	0.001**	0.700	0.761
Bacteria	11.648	<0.001**	19.048	<0.001**	1.024	0.466
Unspecific	2.427	0.09	10.681	<0.001**	1.011	0.477
Total	9.442	<0.001**	19.526	<0.001**	0.967	0.514

*f* value is the value of *F*-test, *Difference was significant at *p* < 0.05, **Difference was significant at *p* < 0.01. SWC, soil water content; TOC, soil total organic carbon; DOC, dissolved organic carbon; TN, total nitrogen; TP, total phosphorus.

### Soil properties and co-occurrence network change with depth and altitude

Soil depth significantly affected DOC, TN, and pH (Table 1). As soil depth increased, DOC decreased and TN generally increased. No significant difference was found in SWC, TOC, and TP between different depths. Altitude significantly affected SWC (Table 1). SWC generally increased with the altitude. No significant difference was found in soil pH, TN, TP, and TOC along the altitudinal gradient.

**Figure 3:**
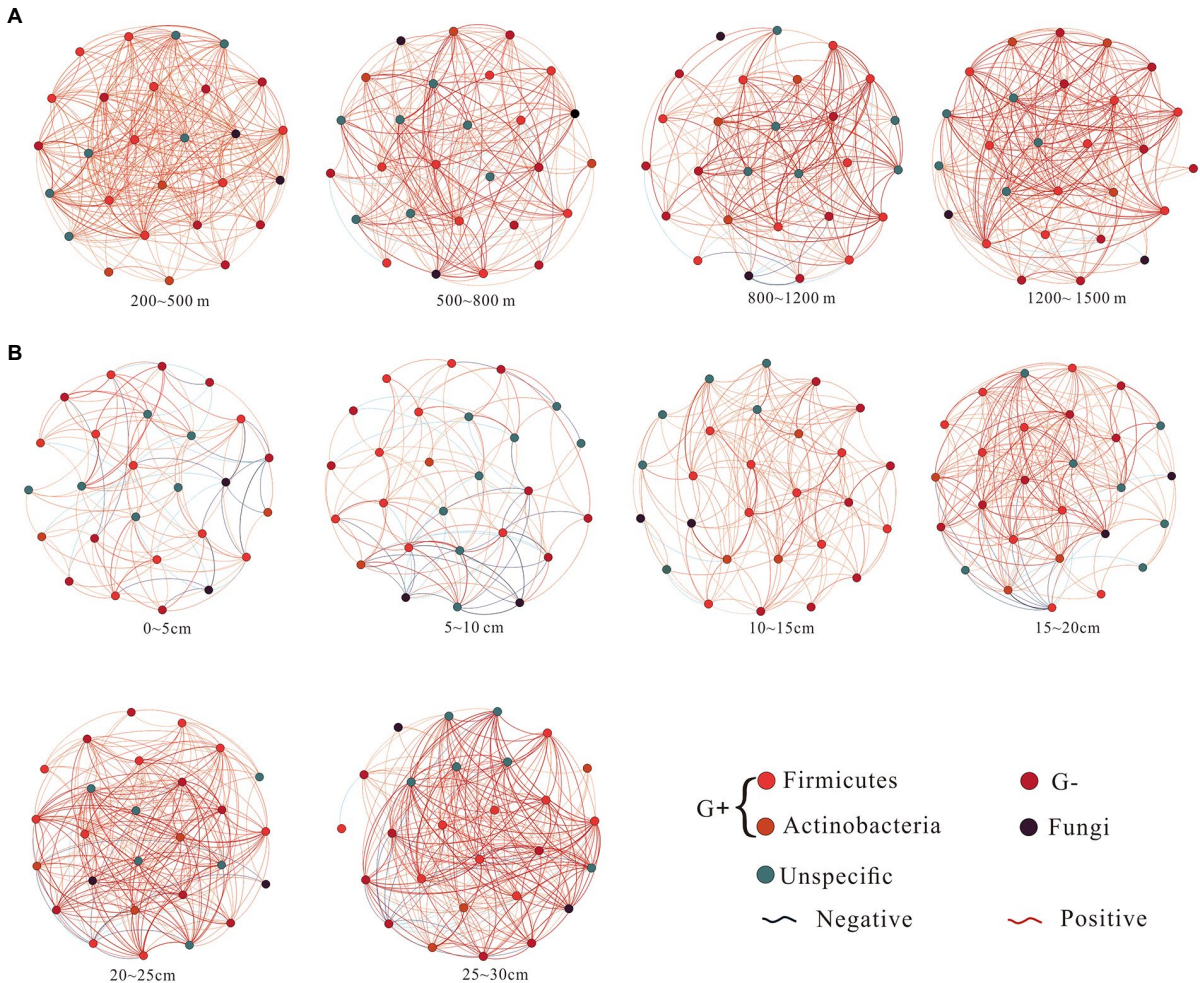
Microbial co-occurrence networks along the altitudinal **(A)** and depth **(B)** gradient on Changbai Mountain, China. The networks of co-occurring microbial biomarkers were determined based on Pearson correlation analysis. The node suggests the individual microbial biomarker based on the phospholipid fatty acid. The co-occurrence network nodes are colored by microbial groups. Blue edges indicate negative relationships between two individual nodes, while red edges suggest positive relationships. A connection stands for a strong correlation coefficient (*r*) >0.5. Each depth network was constructed from eight samples. Each altitudinal network was constructed from 12 samples.

Microbial co-occurrence network did not show significant differences along the altitudinal gradient (Figure 3A), but it had significant differences between soil depths (Figure 3B). As soil depth increased, the negative interaction ratio and average path length of co-occurrence networks decreased, and the positive interaction ratio, average degree, average clustering coefficients, and graph density of co-occurrence networks gradually increased (Table 2; Figure 3B).

**Table 2:** Topological parameters of network analysis in different altitudes and soil depths.

Network attributes	Altitude (m)				Soil depth (cm)					
	200–500	500–800	800–1,200	1,200–1,500	0–5	5–10	10–15	15–20	20–25	25–30
Nodes	27	27	27	27	26	26	27	27	27	27
Edges	267	204	200	240	124	116	168	212	250	252
Interaction positives	100%	96.08%	95.5%	98.75%	79.03%	73.27%	98.21%	94.34%	95.2%	93.65%
Interaction negatives	0%	3.92%	4.5%	1.25%	20.97%	26.73%	1.79%	5.66%	4.8%	6.35%
Average degree	9.889	7.556	7.407	8.889	4.769	4.462	6.222	7.852	9.259	9.333
Modularity	0.073	0.104	0.164	0.111	0.293	0.264	0.186	0.088	0.072	0.046
Graph density	0.38	0.291	0.285	0.342	0.191	0.178	0.239	0.302	0.356	0.359
Average clustering coefficient	0.442	0.368	0.352	0.411	0.296	0.250	0.338	0.389	0.418	0.437
Average path length	1.207	1.336	1.305	1.328	1.542	1.652	1.48	1.394	1.213	1.204

### Relationship between the microbial abundance and biotic and abiotic factors

**Figure 4:**
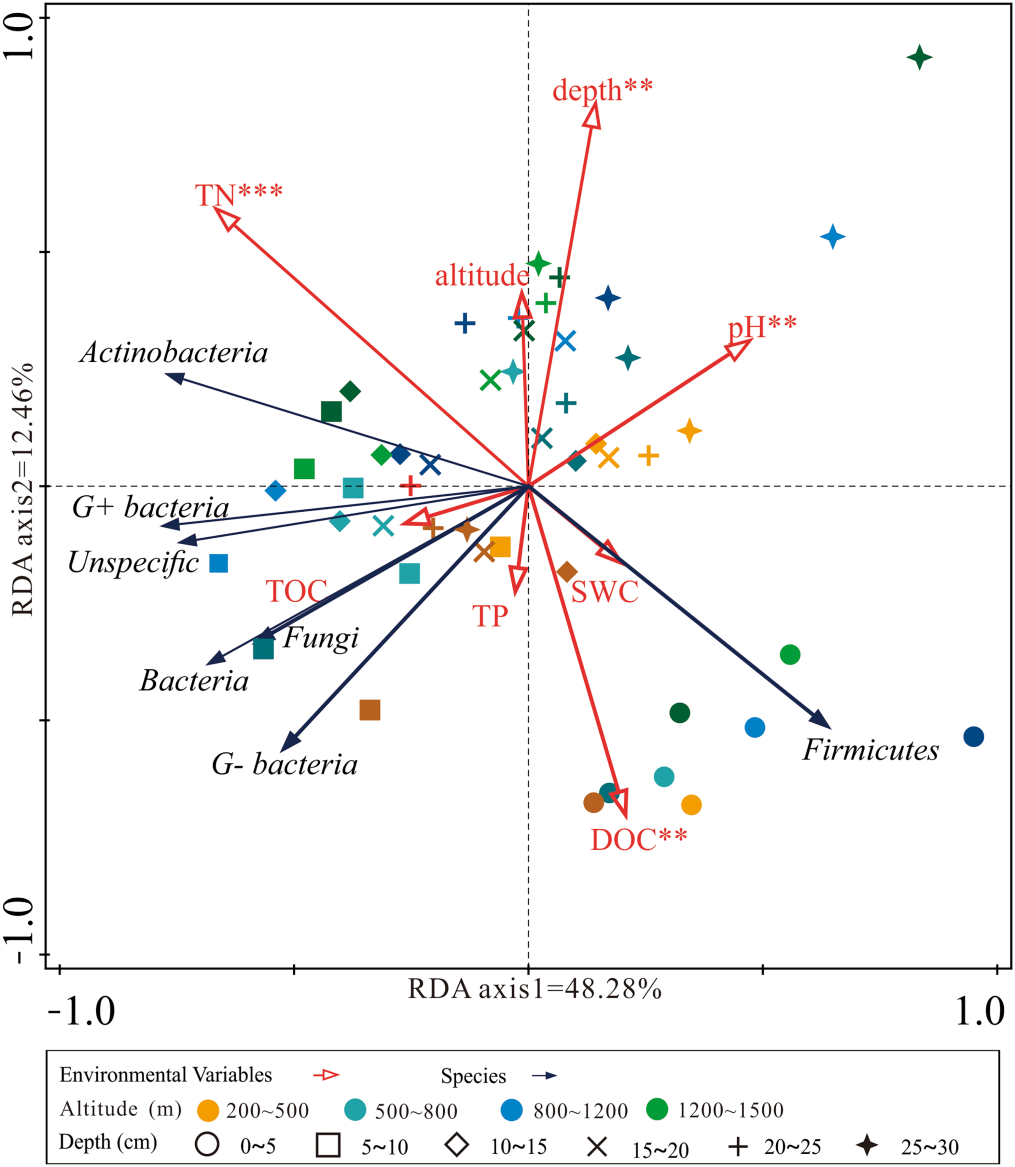
Redundancy analysis (RDA) ordination plot of the concentration of microbial groups constrained by the altitude, soil depth, and physicochemical properties. DOC, dissolved organic carbon; TOC, soil total organic carbon; TP, total phosphorus; TN, total nitrogen; SWC, soil water content.

The RDA analysis found that the first and second axes together explained over 60.0% of the total variation in microbial groups (Figure 4). Soil pH, TN, DOC, and depth were the main factors that significantly affected the microbial abundance. Samples from the top soil layers were located in the lower quadrant of the RDA graph while samples from deeper soil layers were located in the upper quadrant (Figure 4).

**Figure 5:**
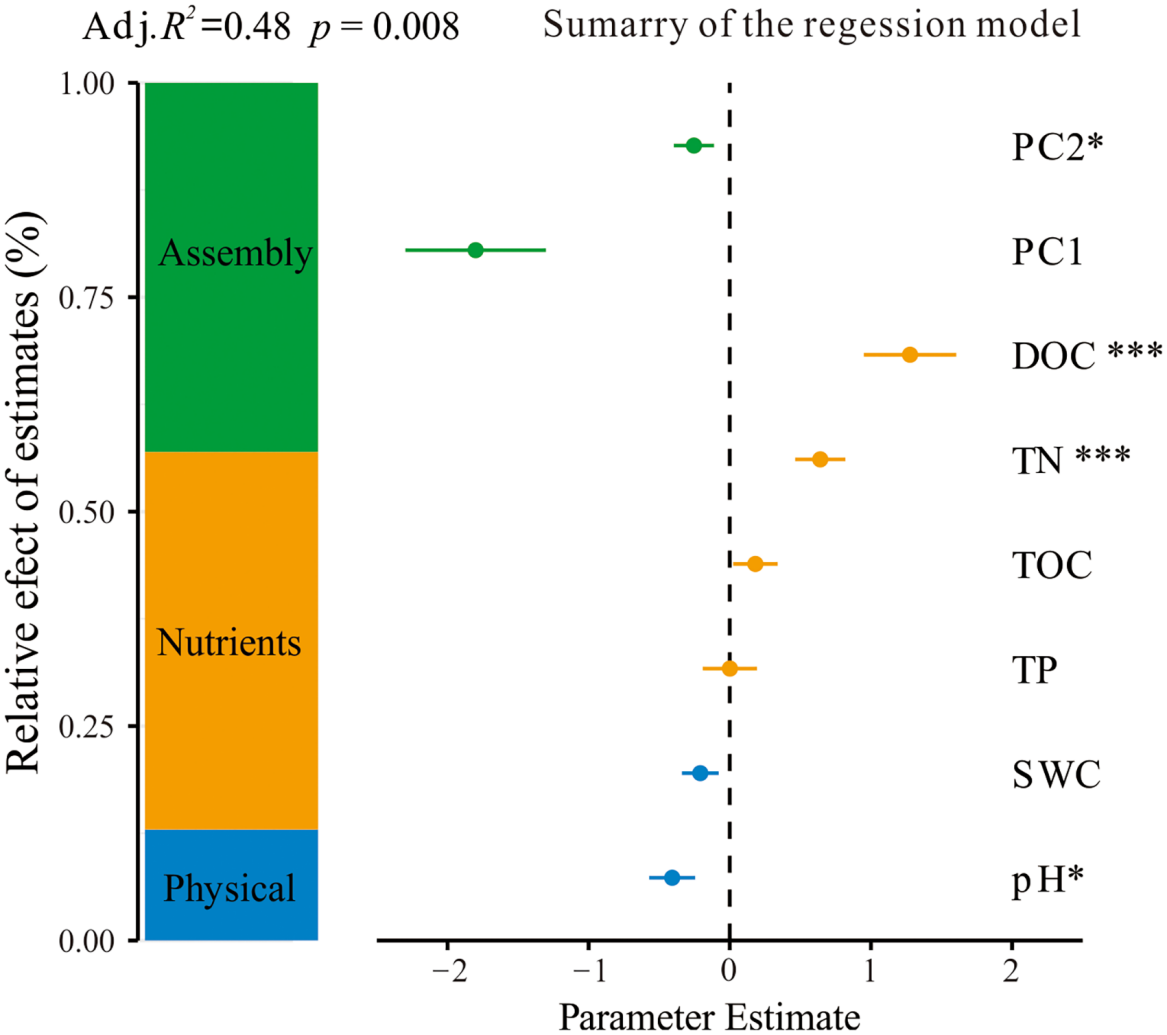
Effect of abiotic and biotic factors on total microbial abundance. Average parameter estimates (standardized regression coefficients) of model predictors, associated 95% confidence intervals, and relative importance of each factor, expressed as the percentage of explained variance. The adjusted (adj.) *R*^2^ of the averaged model and the *p*-value of each predictor were given as: *p < 0.05; **p < 0.01; ***p < 0.001. TOC, soil total organic carbon; DOC, dissolved organic carbon; TN, total nitrogen; TP, total phosphorus; SWC, soil water content. PC1 and PC2, the first and two axes’ scores of the principal component analysis for microbial community composition.

The VDA analysis showed that the total microbial abundance was significantly affected by both abiotic and biotic factors. Soil nutrients including DOC and TN had a positive correlation with the total microbial abundance. Soil physical properties (pH and SWC) and the microbial interaction network had a negative relationship with the total microbial abundance (Figure 5).

## Discussion

### Soil physicochemical properties differed between depths and significantly affected microbial abundance in peatlands

Soil depth has a great impact on soil microbes because of the unequal distribution of plant roots and soil nutrients across soil profiles (Rousk et al., 2010). Soil nutrients and physical environments were the main factors affecting the total microbial abundance in peatlands. Soil nutrients provided energy for the growth, metabolism, and reproduction of microorganisms (Zhang et al., 2022). In our study, soil depth significantly affected soil physicochemical properties and microbial abundance in peatlands. Soil nutrients had a positive influence on the soil microorganisms and soil physical factors had a negative impact on the abundance of soil microbes (Figure 5).

Soil nutrient is a key factor affecting microbial abundance in peatlands. DOC can be used as a carbon substrate for soil microbes, and TN can alleviate carbon limitation on soil microorganisms (Guo et al., 2011; Zhou et al., 2017). In our study, DOC and TN differed significantly between soil depths, and they significantly affected the total microbial abundance (Table 1; Figure 5). This result was consistent with former research (Bradley et al., 2006; Jia et al., 2020; Zhao M. L. et al., 2021). DOC is the most active intermediate in carbon cycle process because of its high mobility and bioavailability (Marschner and Kalbitz, 2003). DOC is utilized as a substrate, leading to microbial mineralization and CO_2_ emissions in peatlands (Battin et al., 2008; Zhang et al., 2022). Nitrogen accumulation increases microbial abundance because it increases the utilization rate of nitrogen resources, which can alleviate carbon limitation on soil microorganisms or inhibit the limitation of carbon caused by soil acidification (Guo et al., 2011; Zhou et al., 2017). This finding indicated that nitrogen accumulation had a positive effect on microbial abundance. The terrestrial surface temperature is projected to exceed 2°C by the end of this century, and the atmospheric nitrogen deposition and the intensity of extreme precipitation events will increase (IPCC, 2022). These changes may lead to more DOC exports from the peatlands (Cole et al., 2002; Clark et al., 2010) and an increasing nitrogen accumulation in peatlands (Zhang et al., 2018, 2022). Our findings suggest that DOC and TN positively affect soil microbial abundance and microbial activities, which may lead to higher CO_2_ emissions in peatlands.

Soil physical properties also affect soil microbial abundance. Soil pH changed cell membrane charge and thus influenced the nutrient absorption by soil microorganisms and the enzyme activity in metabolic processes (Rousk et al., 2010). The acidic environment in peatlands is conducive to the growth of microorganism, and the microbial abundance generally decreased as soil pH increased in our study, which is consistent with former research (Anderson et al., 2010). Water regime could also affect soil microbial abundance. The effects of water drainage on soil microbial community and enzyme activity were dependent on soil depth in peatlands (Xu et al., 2021). When SWC was high, soil microbial abundance decreased because microbial heterotrophic respiration of microorganisms was inhibited (Wagner et al., 2015).

### The complexity of the co-occurrence network increased with soil depths and affected microbial abundance in peatlands

The complexity of the co-occurrence network indicated microbial interactions along the environmental gradient (Ma et al., 2020). Environmental variables are essential for microbial niche differentiation, which enables distinct microbial groups to obtain adequate substrate and survive under various environmental conditions (Wiens et al., 2010; Li et al., 2020). The wide range of edaphic environments has driven the assembly process of soil microorganism (Dini-Andreote et al., 2015; Tripathi et al., 2018). In our study, the complexity of microbial co-occurrence networks increased and the microbial abundance decreased with soil depth (Table 2), and the VDA analysis indicated that microbial community assembly was one major factor affecting microbial abundance (Figure 5). The availability of carbon, energy and oxygen decreased with soil depth in peatlands, the competition of microorganisms for the resource increased, and their interactions increased. Resource limitation leads to the reduction of microbial abundance (Lu et al., 2020). Our results are consistent with a recent study which found the depth effect on bacterial community assembly processes in paddy soils (Li W. T. et al., 2022).

## Conclusion

We explored the change in microbial abundance in peatlands along the depth and altitudinal gradients on Changbai Mountain, China. Soil microbial abundance was more affected by soil depth than the altitude. The microbial abundance at 5–10 cm was higher than that at depth of 0–5 cm and 10–30 cm. The microbial abundance at 500–800 m was higher than that at altitude of 200–500 m and 800–1,500 m. The change in total microbial abundance was driven by both soil physiochemical properties and microbial co-occurrence network. Our study provides a new insight into the significance of microbial participation in peatland carbon cycling along the environmental gradient. It is important to consider the depth effects on soil microbial abundance when assess the peatland carbon dynamic under climate change.

## Data availability statement

The original contributions presented in the study are included in the article/supplementary material, further inquiries can be directed to the corresponding author.

## Author contributions

MZ, MW, and GW designed the study. MZ, MW, YZ, NH, LQ, ZR, and MJ performed the field investigation and collected the data. MZ and GW conducted the statistical analysis and wrote the manuscript. All authors contributed to the article and approved the submitted version.

## Funding

This study was supported by the National Natural Science Foundation of China (42077070, 41877075, 41871081, and U19A2042), the Strategic Priority Research Program of the Chinese Academy of Sciences (XDA28080300), the Professional Association of the Alliance of International Science Organizations (ANSO-PA-2020-14), and the Youth Innovation Promotion Association CAS (2019234 and 2020237).

## Conflict of interest

The authors declare that the research was conducted in the absence of any commercial or financial relationships that could be construed as a potential conflict of interest.

The reviewer FL declared a shared affiliation with the authors MZ, YZ, NH, LQ, ZR, GW, and MJ to the handling editor at the time of review.

## Publisher’s note

All claims expressed in this article are solely those of the authors and do not necessarily represent those of their affiliated organizations, or those of the publisher, the editors and the reviewers. Any product that may be evaluated in this article, or claim that may be made by its manufacturer, is not guaranteed or endorsed by the publisher.
